# Geriatric Conditions in Acutely Hospitalized Older Patients: Prevalence and One-Year Survival and Functional Decline

**DOI:** 10.1371/journal.pone.0026951

**Published:** 2011-11-14

**Authors:** Bianca M. Buurman, Jita G. Hoogerduijn, Rob J. de Haan, Ameen Abu-Hanna, A. Margot Lagaay, Harald J. Verhaar, Marieke J. Schuurmans, Marcel Levi, Sophia E. de Rooij

**Affiliations:** 1 Section of Geriatric Medicine, Department of Internal Medicine, Academic Medical Center, University of Amsterdam, Amsterdam, The Netherlands; 2 Research Group Care for the Chronically Ill, Faculty of Health Care, University of Applied Sciences Utrecht, Utrecht, The Netherlands; 3 Clinical Research Unit, Academic Medical Center, University of Amsterdam, Amsterdam, The Netherlands; 4 Department of Medical Informatics, Academic Medical Center, University of Amsterdam, Amsterdam, The Netherlands; 5 Department of Internal Medicine, Spaarne Hospital, Hoofddorp, The Netherlands; 6 Department of Geriatrics, University Medical Center Utrecht, Utrecht, The Netherlands; 7 Department of Nursing Science, University Medical Center Utrecht, Utrecht, The Netherlands; Asociacion Civil Impacta Salud y Educacion, Peru

## Abstract

**Background:**

To study the prevalence of eighteen geriatric conditions in older patients at admission, their reporting rate in discharge summaries and the impact of these conditions on mortality and functional decline one year after admission.

**Method:**

A prospective multicenter cohort study conducted between 2006 and 2008 in two tertiary university teaching hospitals and one regional teaching hospital in the Netherlands. Patients of 65 years and older, acutely admitted and hospitalized for at least 48 hours, were invited to participate. Eighteen geriatric conditions were assessed at hospital admission, and outcomes (mortality, functional decline) were assessed one year after admission.

**Results:**

639 patients were included, with a mean age of 78 years. IADL impairment (83%), polypharmacy (61%), mobility difficulty (59%), high levels of primary caregiver burden (53%), and malnutrition (52%) were most prevalent. Except for polypharmacy and cognitive impairment, the reporting rate of the geriatric conditions in discharge summaries was less than 50%. One year after admission, 35% had died and 33% suffered from functional decline. A high Charlson comorbidity index score, presence of malnutrition, high fall risk, presence of delirium and premorbid IADL impairment were associated with mortality and overall poor outcome (mortality or functional decline). Obesity lowered the risk for mortality.

**Conclusion:**

Geriatric conditions were highly prevalent and associated with poor health outcomes after admission. Early recognition of these conditions in acutely hospitalized older patients and improving the handover to the general practitioner could lead to better health outcomes and reduce the burden of hospital admission for older patients.

## Introduction

Approximately ten percent of people over 65 years old are admitted to the hospital annually, and these patients' hospital visits account for half of all days spent in the hospital [1]. Acute illness leading to hospital admission is often accompanied by multiple chronic diseases and conditions such as decreased ability to perform Activities of Daily Living (ADL), cognitive impairment, delirium, falls and malnutrition [2]–[7]. The clinical importance of these health problems, in this article defined as geriatric conditions, should not be underestimated because their presence reflects reduced functional and physiological reserves [8], [9]. The combination of diseases and geriatric conditions is an important predictor of adverse events during hospital admission, and it is also associated with functional and cognitive decline, institutionalization and mortality after discharge [8]–[13].

The clinical relevance of screening for geriatric conditions at hospital admission not only involves decisions, such as preventing or actively treating geriatric conditions, during and after the hospital stay, but also contributes to decisions of whether or not to begin with invasive treatment for the acute illness. From a patient perspective, information on the presence of geriatric conditions and their negative impact on health outcomes assists patients and their primary caregivers in making a well-informed decision concerning preferred treatment goals. Moreover, some studies have shown that, in specific patient populations, early recognition of geriatric conditions by means of a comprehensive geriatric assessment (CGA) might reduce adverse events during and after hospital stay [14]–[16]. Early recognition of geriatric conditions may also contribute to better health status after hospitalization in terms of functional and cognitive abilities [16]–[18].

Despite this knowledge, current medical practice in hospitals is mainly performed according to the traditional disease model of medicine, which mainly focuses on the presence of diseases, and geriatric conditions may be overlooked or ignored in the care of older patients. We performed a prospective cohort study on acutely hospitalized older persons to investigate the prevalence of geriatric conditions at hospital admission, the reporting rate of these geriatric conditions in discharge summaries and to assess the impact of geriatric conditions on one-year health outcomes in terms of mortality and functional decline.

## Methods

### Design and setting

This multicenter prospective cohort study, the DEFENCE (Develop strategies Enabling Frail Elderly New Complications to Evade) study, was conducted between April 1, 2006 and April 1, 2008 in eleven general internal medicine wards in three hospitals in The Netherlands: the Academic Medical Center (AMC) in Amsterdam; the University Medical Center Utrecht (UMCU) in Utrecht; and the Spaarne Hospital (SH) in Hoofddorp. The AMC (1024 beds) and UMCU (1042 beds) are tertiary university teaching hospitals. The SH (455 beds) is a large regional teaching hospital.

In total, five wards in the AMC, three wards in the UMCU and three wards in the SH participated. The staff on the general medical wards consisted of residents, physicians and registered nurses. They were not specialists trained in geriatric medicine or geriatric nursing. All hospitals had a geriatric consultation team available that consisted of at least one clinical nurse specialist in geriatrics and one geriatrician.

The study was approved by the Medical Ethics Committee of the AMC. Local approval was given by the UMCU and SH.

### Patients

All patients aged 65 years and older who were acutely admitted to one of the general internal medical wards of the three hospitals were enrolled in the study. Patients were excluded because of any of the following: 1) they or their relatives did not provide informed consent, 2) they were unable to speak or understand Dutch, 3) they came from another ward inside or outside the hospital, 4) they were transferred to the Intensive Care Unit, the Coronary Care Unit or another ward in or outside the hospital within 48 hours of admission or 5) they were terminally ill. Patients had to be enrolled in the study within 48 hours of hospital admission, and written informed consent was obtained prior to enrollment.

### Data collection

A research nurse visited the participating wards on a daily basis (except for the weekends) to identify eligible patients for the study. After the patient's written informed consent (or that of the patient's primary caregiver in case of cognitive impairment) was obtained, the patient received a systematic comprehensive geriatric assessment (CGA), which was administered by a research nurse. The CGA of the patient had to be completed within 48 hours of admission. The primary caregiver was also interviewed. The primary caregiver is defined as a family member or other person who received no formal training who provide daily care on a voluntary basis to the patient that is admitted to the hospital. To obtain uniformity in conducting the CGA, the research nurses were trained in interviewing patients and primary caregivers before the start of the study, and ten patients were assessed simultaneously by the research nurse and geriatrician to control for observer variability.

### Comprehensive Geriatric Assessment of geriatric conditions

The CGA in the current study consisted of a systematic assessment of geriatric conditions and focused on four domains of the patient's function (somatic, psychological, functional and social). The CGA evaluated 18 health problems that are frequently observed in older persons, defined in this article as geriatric conditions. Table S1 shows the content of the CGA, including applied measurement instruments, score ranges and the cut-off scores used. Data collection began with the 11-item Mini-Mental State Examination (MMSE), to assess the presence and degree of global cognitive impairment [19]. Patients with a MMSE score of ≥21 points were interviewed. The responses of patients with a MMSE score of 16–20 points, indicating moderate global cognitive impairment, were cross-checked with those of their primary caregiver concerning baseline characteristics and ADL performance. In case of a disagreement, the response of the primary caregiver was selected. Data from patients with a MMSE score of ≤15 points were obtained from their primary caregiver. This latter group was not screened for pain or depressive symptoms, as the measurement instruments we used have not been validated in cognitively impaired patients.

After enrolling a patient and completing the main part of the CGA, the research nurse reported her findings to the geriatrician. The geriatrician also visited the patient within 48 hours and paid special attention to evaluating potential psychiatric problems, such as delirium. The patient was screened for delirium using the Confusion Assessment Method (CAM) [20].

After discharge, a geriatrician studied the discharge letter on medical diagnoses present at admission, new conditions that developed during the hospital stay, co-morbidities and medication. The Charlson co-morbidity index was also derived from this information [21], indicating the number and severity of co-morbidities. The possible scores on the Charlson co-morbidity index range from 0 to 31, with a higher score indicating a greater number of co-morbidities and/or more severe co-morbidities. The ICD-9 diagnostic criteria were used to determine the presence of all medical diagnoses.

The geriatrician also scored if the discharge summary contained direct or indirect information on the presence of the eighteen geriatric conditions at discharge. This latter part was only done with patients from the AMC. To get uniformity in this screening process, three geriatricians were invited to discuss the direct and indirect information that could indicate the presence of the geriatric condition. Direct information was the statement of the geriatric condition in the discharge summary; indirect information concerned for example, information on not eating of losing weight before or during hospital admission or dietary advice at follow up, in case of malnutrition.

### Follow-up and outcome assessments

One year after admission, a research nurse examined the municipal data registry to determine whether patients were alive. The exact date of death was registered if a patient had died. The nurse then contacted all other patients and their primary caregivers by telephone to assess the patients' present functional status, in terms of functional decline and cognitive impairment. All outcome data were collected from the same person (patient or primary caregiver) who responded at baseline.

Functional decline was defined as a loss of at least one point on the original Katz ADL index score [22] one year after hospital admission compared to the premorbid Katz ADL index score, which was assessed based on patients' performance two weeks prior to hospital admission.

Cognitive impairment one year after hospital admission was defined as a score of 3.9 or more on the Informant Questionnaire on Cognitive Decline in the Elderly-Short Form (IQCODE-SF), which was completed by the primary caregiver of the patient [23].

### Statistical analysis

Patient and clinical baseline characteristics, the prevalence of geriatric conditions and health outcomes were summarized using descriptive statistics. Because the dataset suffered from missing data on the independent variables (geriatric conditions), we performed multiple imputation as implemented by SPSS, version 18.0.2. In this approach, all geriatric conditions were entered into the imputation model (predictor and imputation, together with sex, age, the Charlson co-morbidity score and mortality and functional decline (predictors only). Five imputation datasets were used. Because depression and pain were not systematically assessed in severely cognitive impaired patients, these geriatric conditions were not imputated and were not analyzed further in the regression models.

The independent impact of geriatric conditions on mortality and poor outcome (mortality or functional decline) was analyzed using Cox regression models. Geriatric conditions with a p<0.20 in the univariable analyses were entered into the multivariable models. In these multivariable models sex, age and Charlson comorbidity scores, were also entered as independent predictors as these variables are known risk factors for mortality. Effect sizes were expressed in hazard ratios with their corresponding 95% confidence intervals. The Cox models were checked for collinearity between independent variables; the proportional hazards assumption was verified using log-minus-log plots.

The analysis for functional decline excluded patients who died and those with a maximum score on the premorbid Katz ADL index because there was no room for further decline within these patients. Logistic regression analysis was conducted and effect sizes were expressed in odds ratios with their corresponding 95% confidence intervals. Geriatric conditions with a p<0.20 in the univariable analyses were entered into the multivariable models. The logistic regression models were checked for collinearity between independent variables.

## Results

### Participants

There were 1031 consecutive patients eligible for participation in this study, of whom 639 (62%) were enrolled after providing informed consent. The reasons for exclusion were refusal to participate (n = 222), insufficient Dutch language capacities (n = 86), transfer from another ward (n = 36), transfer to another ward such as an ICU or CCU within 48 hours (n = 28) and terminal illness (n = 20). Follow-up concerning mortality was completed for 100% of the patients. Functional and cognitive outcome was completed for 92% and 77% of included patients, respectively. Compared to included patients, excluded patients were significantly younger (75 years vs. 78 years, p<0.001) and died more frequently within one year of discharge (48% vs. 35%, p<0.001).

### Baseline characteristics and prevalence of geriatric conditions

Table 1 presents the baseline characteristics of the study population. The mean age was 78 years, and 72% of patients lived independently prior to hospital admission. The primary reason for hospital admission was infectious disease (41%). The mean (SD) number of geriatric conditions at hospital admission was six (3). Table 2 shows the prevalence of each geriatric condition. Overall, impairment in Instrumental Activities of Daily Living (IADL) (83%), polypharmacy (61%), mobility difficulties (59%), perceived burden on caregivers (53%), malnutrition (52%) and ADL impairments were the most common geriatric conditions. Cognitive impairment at admission was present in 40% of study patients; of these patients, 6% had a diagnosed dementia that was reported in the hospital file. All conditions were apparent in at least 13% of the patients, except for pressure ulcers (4%). Table 2 also provides information on the total number of observations that were present before the imputation of the dataset.

**Table 1:** Baseline Characteristics of Patients.

Variable	Total group(n = 639)
**Age in years, mean (SD)**	78.2 (7.8)
**Sex**	
Male, % (No.)	46.2 (295)
**Ethnicity**	
Caucasian, % (No.)	92.8 (593)
**Living arrangement**	
Independent, % (No.)	72.4 (463)
**Social status**	
Living with partner or child, % (No.)	47.9 (306)
**Education in years, mean (SD)**	9.9 (3.9)
**Medical reason for admission, %, (No.)**	
Infectious disease	40.9 (261)
Gastrointestinal disease	22.8 (146)
Malignancy	6.2 (40)
Cardiovascular disease	4.3 (27)
Water and electrolyte disturbance	10.5 (67)
Other	15.4 (98)
**Charlson comorbidity index score** * **, mean (SD)**	3.7 (2.4)

*Range 0–31; a higher score indicates more and/or more severe comorbidities.

**Table 2:** Prevalence of Geriatric Conditions in Acutely Hospitalized Older Patients.

Geriatric condition	Overall prevalence, % (Number of patients/total number of observations)(n = 639)
**Somatic domain**	
Polypharmacy	60.7 (386/636)
Malnutrition	51.5 (322/635)
Obesity	13.5 (77/569)
Pain*	42.6 (206/483)
Fall risk	23.3 (142/609)
Pressure ulcer	3.6 (19/582)
Indwelling urinary catheter	23.8 (150/631)
Incontinence	22.2 (137/618)
Constipation	19.5 (123/630)
**Psychological domain**	
Cognitive impairment	40.1 (256/639)
Depressive symptoms*	21.1 (101/479)
Delirium	19.0 (118/622)
**Functional domain**	
Premorbid ADL impairment	50.9 (324/637)
Premorbid IADL impairment	82.5 (527/639)
Vision impairment	22.3 (137/613)
Hearing impairment	20.3 (120/590)
Mobility difficulty	58.5 (373/638)
**Social**	
High perceived burden on caregivers	52.7 (267/507)

ADL = activities of daily living, IADL = instrumental activities of daily living.

*Only assessed in patients with MMSE≥16 (n = 483).

### Reporting rate of geriatric conditions in discharge summaries

The reporting rate of each of the geriatric conditions was low (Table 3). As expected, the presence of five or more different medications was always mentioned. Cognitive impairment (54%) and delirium during hospital stay (50%) were both reported in half of the times they were present at hospital admission, followed by pain (38%) and malnutrition (22%). Some geriatric conditions were never mentioned, such as pressure ulcers and hearing impairment.

**Table 3:** Reporting rate of geriatric conditions screened at admission in discharge summaries.

Geriatric condition	Reporting rate if geriatric condition was present (%)(n = 407)
**Somatic domain**	
Polypharmacy	100.0
Malnutrition	22.2
Obesity	NA
Pain	38.0
Fall risk	19.9
Pressure ulcer	0.0
Indwelling urinary catheter	NA
Incontinence	1.4
Constipation	13.6
**Psychological domain**	
Cognitive impairment	54.0
Depressive symptoms	1.8
Delirium	50.0
**Functional domain**	
Premorbid ADL impairment	8.1
Premorbid IADL impairment	NA
Vision impairment	1.2
Hearing impairment	0.0
Mobility difficulty	8.8
**Social domain**	
High perceived burden on caregivers	7.7

**NA = not available.**

### Follow-up

Impaired outcomes were common one year after admission. The mortality rate one year after hospital admission was 35%. Of those patients who were alive after one year, 33% encountered functional decline and 26% experienced cognitive impairment. Overall, 54% exhibited a poor outcome in terms of mortality or functional decline.

Table 4 shows the Cox regression models for mortality and functional decline one year after admission. Male sex, a higher score on the Charlson comorbidity index and the geriatric conditions of malnutrition, fall risk, delirium and IADL impairment had a significant impact on higher mortality rates. Obesity was associated with lower mortality. The analysis for poor outcome (functional decline including mortality) partly showed same risk profile for the geriatric conditions as compared to mortality only; malnutrition, fall risk, delirium and premorbid ADL impairment were all associated with poor outcome. Older age and higher Charlson comorbidity index were also significantly associated.with poor outcome. In the survivors, older age, an indwelling urinary catheter and IADL impairment had a significant impact on functional decline (Table 5)

**Table 4:** Cox Regression Analyses for Mortality and Poor Outcome One Year after Hospital Admission.

	Mortality				Poor outcome (mortality or functional decline)			
Variables	UnivariableHR (95% CI)	p-value	MultivariableHR (95% CI)	p-value	UnivariableHR (95% CI)	p-value	MultivariableHR (9% CI)	p-value
Male sex	1.49 (1.14 to 1.93)	0.01	1.33 (1.02 to 1.75)	0.04	1.17 (0.94 to 1.44)	0.15	-	-
Age (per year)	1.04 (1.01 to 1.04)	0.01	-	-	1.03 (1.02 to 1.05)	<0.001	1.02 (1.01 to 1.04)	0.01
Charlson comorbidity score (per point)	1.21 (1.15 to 1.28)	<0.001	1.19 (1.13 to 1.26)	<0.001	1.14 (1.10 to 1.20)	<0.001	1.16 (1.11 to 1.21)	<0.001
Polypharmacy	1.04 (0.80 to 1.37)	0.75			1.10 (0.88 to 1.36)	0.42		
Malnutrition	2.18 (1.65 to 2.89)	<0.001	1.80 (1.35 to 2.40)	<0.001	1.55 (1.25 to 1.92)	<0.001	1.41 (1.13 to 1.75)	0.01
Obesity	0.34 (0.18 to 0.64)	0.01	0.50 (0.26 to 0.97)	0.04	0.70 (0.49 to 0.99)	0.05	-	-
Fall risk	1.71 (1.28 to 2.30)	<0.001	1.48 (1.09 to 2.01)	0.01	1.57 (1.23 to 2.00)	<0.001	1.32 (1.03 to 1.70)	0.03
Presence of a pressure ulcer	2.74 (1.46 to 5.17)	0.01	-	-	2.17 (1.20 to 3.92)	0.01	-	-
Indwelling urinary catheter	1.47 (1.10 to 1.97)	0.10	-	-	1.63 (1.30 to 2.05)	<0.001	-	-
Incontinence	1.05 (0.77 to 1.44)	0.74			1.00 (0.77 to 1.30)	0.98		
Constipation	1.25 (0.91 to 1.73)	0.17	-	-	1.13 (0.87 to 1.48)	0.36		
Cognitive impairment (per MMSE point)	0.96 (0.95 to 0.98)	<0.001	-	-	0.96 (0.95 to 0.98)	<0.001	-	-
Prevalent delirium	2.10 (1.57 to 2.81)	<0.001	1.46 (1.02 to 2.09)	0.04	1.95 (1.53 to 2.48)	<0.001	1.52 (1.14 to 2.03)	0.01
ADL impairment (per point)	1.21 (1.14 to 1.29)	<0.001	-	-	1.13 (1.08 to 1.20)	<0.001	-	-
IADL impairment (per point)	1.18 (1.12 to 1.24)	<0.001	1.13 (1.06 to 1.19)	<0.001	1.15 (1.11 to 1.20)	<0.001	1.08 (1.03 to 1.14)	0.01
Vision impairment	0.94 (0.67 to 1.30)	0.70			1.05 (0.81 to 1.35)	0.73		
Hearing impairment	1.24 (0.90 to 1.72)	0.19	-	-	1.26 (0.97 to 1.64)	0.08	-	-
Mobility difficulty	1.44 (1.10 to 1.89)	0.10	-	-	1.60 (1.28 to 1.99)	<0.001	-	-

Field marked with a – indicates that the variable had a p<0.20 in the univariable Cox regression analysis and was entered into the multivariable Cox regression model.

HR = Hazard Ratio, CI = confidence interval.

MMSE = Mini-Mental State Examination, range of scores between 0–30, a higher score indicates better global cognitive functioning.

ADL = activities of daily living, range of scores between 0–6, a higher score indicates more dependence in ADL, IADL = instrumental activities of daily living, range of scores between 0–8, a higher score indicates more dependence in IADL.

Functional decline was defined as a loss of at least 1 point on the Katz ADL index score one year after admission compared to premorbid functioning two weeks prior to hospital admission.

**Table 5:** Logistic regression analysis for functional decline one year after admission in acutely hospitalized older patients.

	Functional decline			
Variables	UnivariableOR (95% CI)	p-value	MultivariableOR (95% CI)	p-value
Male sex	0.69 (0.44 to 1.08)	0.10	-	-
Age (per year)	1.07 (1.04 to 1.11)	<0.001	1.05 (1.02 to 1.09)	0.01
Charlson comorbidity score (per point)	1.04 (0.93 to 1.16)	0.52		
**Geriatric conditions**				
**Somatic domain**				
Polypharmacy	1.43 (0.92 to 2.24)	0.12	-	-
Malnutrition	0.84 (0.54 to 1.31)	0.45		
Obesity	1.55 (0.85 to 2.81)	0.16	-	-
Fall risk	1.68 (0.94 to 2.98)	0.08	-	-
Presence of a pressure ulcer	1.80 (0.14 to 24.13)	0.65		
Indwelling urinary catheter	2.97 (1.74 to 4.92)	<0.001	2.15 (1.24 to 3.72)	0.01
Incontinence	1.15 (0.66 to 2.00)	0.62		
Constipation	0.92 (0.52 to 1.65)	0.79		
**Psychological domain**				
Cognitive impairment (per MMSE point)	0.94 (0.90 to 0.97)	<0.001	-	-
Prevalent delirium	2.47 (1.34 to 4.54)	0.01	-	-
**Functional domain**				
ADL impairment (per point)	1.04 (0.88 to 1.22)	0.66		
IADL impairment (per point)	1.26 (1.14 to 1.39)	<0.001	1.17 (1.05 to 1.30)	0.01
Vision impairment	1.46 (0.87 to 2.45)	0.16	-	-
Hearing impairment	1.48 (0.83 to 2.64)	0.19	-	-
Mobility difficulty	2.67 (1.69 to 4.23)	<0.001	-	-

Field marked with a – indicates that the variable had a p<0.20 in the univariable logistic regression analysis and was entered into the multivariable logistic regression model.

OR = Odds Ratio, CI = confidence interval.

MMSE = Mini-Mental State Examination, range of scores between 0–30, a higher score indicates better global cognitive functioning.

ADL = activities of daily living, range of scores between 0–6, a higher score indicates more dependence in ADL, IADL = instrumental activities of daily living, range of scores between 0–8, a higher score indicates more dependence in IADL.

Functional decline was defined as a loss at least of 1 point on the Katz ADL index score one year after admission compared to premorbid functioning two weeks prior to hospital admission.

Patients who died or had a maximum score on the Katz ADL index at admission were excluded from the analysis.

## Discussion

This multicenter prospective cohort study demonstrated that geriatric conditions are highly prevalent in acutely hospitalized older medical patients, Older patients presented with an average of six geriatric conditions. IADL impairment, polypharmacy, mobility difficulty, high levels of perceived caregiver burden, malnutrition and ADL impairment were all present in more than 50% of the patients. Premorbid IADL impairment was associated with all negative health outcomes, whereas the presence of malnutrition, fall risk and delirium were associated with mortality and the composite endpoint poor outcome.

The systematic screening procedure to identify geriatric conditions revealed that the prevalence of geriatric conditions in this patient group is high. The prevalence of some conditions, such as malnutrition [6], cognitive impairment [4], [5], delirium [3], [24], (I)ADL impairment [2], [8], [25], incontinence [26] and visual impairment [27], is comparable to the prevalence rates reported in other studies of acutely hospitalized older patients. For many of these geriatric conditions, (preventive) interventions can be initiated at the time of hospital admission, which might lead to better health outcomes, as has been demonstrated for the delirium [28], incontinence [29], and malnutrition [30]. Compared to community-dwelling older patients, the prevalence rates for individual geriatric conditions we found in acutely hospitalized older patients are much higher [31].

We also demonstrated that the reporting rate of the geriatric conditions in discharge summaries was low. Although one can argue the relevance of reporting geriatric conditions in the discharge summarie, at least some of the conditions were associated with mortality and functional decline and might not be solved at hospital discharge, such as malnutrition and fall risk. Moreover, patients who had delirium during their hospital stay are at increased risk for a new episode of delirium [3]. This is crucial information for the general practitioner, who is taking over care coordination after discharge. We did not screen the complete medical chart on the recognition of geriatric conditions, but our hypothesis is that the underreporting of geriatric conditions actually reflects the underrecognition of geriatric conditions during hospital stay. This hypothesis has already been confirmed in delirious patients [32], [33].

Mortality one year after admission was substantial, as one-third of the patients died. The presence of malnutrition, fall risk, delirium and IADL dysfunction were significantly associated with higher mortality rates one year after hospital admission. Obesity showed a negative association with mortality, which is difficult to explain based on the literature [34], [35] or clinical reasoning. Malnutrition is a known risk factor for mortality, whereas fall risk and delirium are geriatric conditions that are most often observed in older patients [24], [36], [37]. Analyzing the aggregate outcome of functional decline and mortality results in the same risk profile of poor outcome found when analyzing mortality only.

One year after admission, further functional decline was found in one-third of the survivors. Rates of functional decline after acute hospitalization ranged between 10–50% in the literature [12], [38]. Besides older age, an independent association between an indwelling urinary catheter and impaired functional health was found. The impact of indwelling urinary catheters has been less studied, and these catheters have not been identified as a risk factor for functional decline [39]. This risk factor may be a proxy for acute illness or for patient vulnerability. IADL impairment was associated with all adverse outcomes, and was a more important predictor than premorbid ADL impairment.

Some limitations of the study should be stated. First, a portion of the eligible patients declined to participate in the study. Limitations of this type are frequently encountered in studies of acutely hospitalized older patients and could account for lower inclusion rates than in other studies involving older patients [40]. Compared to some randomized clinical trials, the current study achieved higher rates of inclusion and had lower drop-out rates [15], [41], [42]. The inclusion rate might have implications for the generalizability of the study results. We demonstrated that the patients who were not included were significantly younger and had a higher mortality rate within one year. Furthermore, the research team did not record the invasive diagnostic and treatment procedures patients received during their hospital stay. These procedures could have affected negative health outcomes after discharge as well as new episodes of illness in the year after hospital admission.

The strength of the present study is that it was a representative multicenter study that included many patients with mild to severe cognitive impairment, a group of patients that is often excluded from studies despite being at high risk for many negative outcomes. Approximately 40% of the included patients presented with cognitive impairment, partly due to delirium, or had concentration and memory problems at admission caused by the acute illness. The majority of the eighteen conditions were diagnosed by questioning the primary caregiver, screening the patient or relying on observations made by the research nurse.

The present study might have clinical implications for the care provided in hospitals and after discharge. Many geriatric conditions associated with poor health outcomes require extra attention to prevent further decline and poor outcome. A systematic assessment of geriatric conditions at the time of hospital admission should, therefore, result in an appropriate treatment plan with realistic goals. Treatment goals should be tailored to the individual needs of patients. This strategy might vary from prevention and improving physical functioning in patients without disabilities to maintaining quality of life in older patients with many comorbidities, disabilities and geriatric conditions [43], [44]. An important discussion is which geriatric condition than should be reported in the discharge summary, to ensure continuation of this information in a care plan in primary care.

In conclusion, the current study demonstrates that acutely hospitalized older patients are a vulnerable patient group. In addition to acute illnesses, these patients often present with many geriatric conditions at hospital admission. Poor outcomes, in terms of mortality and functional decline were substantial and were associated with geriatric conditions. Proactive recognition of these conditions and better incorporating this information in the discharge summary might lead to better health outcomes and reduce the burden of hospital admission for older patients.

## Supporting Information

Table S1 (DOC)
