# Supplementary material for: Geriatric Conditions in Acutely Hospitalized Older Patients: Prevalence and One-Year Survival and Functional Decline
Source: PLoS One. 2011 Nov 14;6(11):e26951. doi: 10.1371/journal.pone.0026951 (PMC3215703; doi:10.1371/journal.pone.0026951)
Supplement: Table S1 — (DOC) [file pone.0026951.s001.doc]

**Supporting information table 1 Content of the Comprehensive Geriatric Assessment**

| **Geriatric condition** | **Measurement instrument** | **Range of scores** | **Cut-off score** |
| --- | --- | --- | --- |
| **Somatic domain** | | | |
| Polypharmacy | Counting the number of different medications (continuous use) | Continuous | ≥5 |
| Malnutrition | Short Nutritional Assessment questionnaire (SNAQ) [45] | 0-7 | ≥2 moderately malnourished  ≥3 severely malnourished |
| Obesity | Body Mass index | Continuous | ≥30 |
| Pain * | Visual analogue scale [46] | 0-10 | ≥4 |
| Fall risk | Have you fallen two or more times in the past three months? [47] | Yes or no | Yes |
| Presence of a pressure ulcer | Observation by the research nurse | Yes or no | Yes |
| Indwelling urinary catheter | Presence of a catheter at admission | Yes or no | Yes |
| Incontinence | Self-report of incontinence for urine or feces at admission | Yes or no | Yes |
| Constipation | Self-report of constipation at admission | Yes or no | Yes |
| **Psychological domain** | | | |
| Cognitive impairment | Mini-Mental State Examination [19] | 0-30 | ≤ 24 is cognitive impairment |
| Depressive symptoms * | Two questions, namely: [48]  1. Did you feel sad, depressed or hopeless in the past month?  2. Did you lose interest in daily activities? | 0-2 | 2 |
| Delirium | Confusion Assessment Method [20] | 0-4 | Item 1 and 2 and item 3 and/or 4 are present |
| **Functional domain** | | | |
| Premorbid ADL functioning | Katz ADL index score [22] | 0-6 | ≥1 |
| Premorbid IADL functioning | IADL questions of the modified Katz ADL index score [49] | 0-8 | ≥1 |
| Vision impairment | Do you have problems with your vision, regardless of the use of glasses? | Yes or no | Yes |
| Hearing impairment | Do you have problems with hearing, regardless of the use of a hearing aid? | Yes or no | Yes |
| Mobility difficulty | Are you using a walking device? | Yes or no | Yes |
| **Social domain** | | | |
| High perceived burden of caregivers | Experienced burden of primary care givers (EDIZ) [50] | 0-9 | ≥4 |

* Only assessed in patients with MMSE ≥ 16

References

45. Kruizenga HM, Seidell JC, de Vet HC, Wierdsma NJ, van Bokhorst-de van der Schueren MA (2005) Development and validation of a hospital screening tool for malnutrition: the short nutritional assessment questionnaire (SNAQ). Clin Nutr 24: 75-82.

46. Collins SL, Moore RA, McQuay HJ (1997) The visual analogue pain intensity scale: what is moderate pain in millimetres? Pain 72: 95-97.

47. Oliver D, Papaioannou A, Giangregorio L, Thabane L, Reizgys K et al (2008) A systematic review and meta-analysis of studies using the STRATIFY tool for prediction of falls in hospital patients: how well does it work? Age Ageing 37: 621-627.

48. Arroll B, Khin N, Kerse N (2003) Screening for depression in primary care with two verbally asked questions: cross sectional study. BMJ 327: 1144-1146.

49. Weinberger M, Samsa GP, Schmader K, Greenberg SM, Carr DB et al (1992) Comparing proxy and patients' perceptions of patients' functional status: results from an outpatient geriatric clinic. J Am Geriatr Soc 40: 585-588.

50. Pot AM, van DR, Deeg DJ (1995) [Perceived stress caused by informal caregiving. Construction of a scale]. Tijdschr Gerontol Geriatr 26: 214-219.
